# The Influence of Biogeographic Diversity, Climate and Wildlife on the Incidence of Tick-Borne Encephalitis in Croatia

**DOI:** 10.3390/v17020266

**Published:** 2025-02-14

**Authors:** Josip Bago, Linda Bjedov, Marko Vucelja, Kristijan Tomljanović, Nataša Cetinić Balent, Sanja Zember, Josip Margaletić, Oktavija Đaković Rode

**Affiliations:** 1 Public Health Institute of Varaždin County, 42000 Varaždin, Croatia; josip.bago@zzjzzv.hr; 2 Faculty of Forestry and Wood Technology, University of Zagreb, 10000 Zagreb, Croatia; lbjedov@sumfak.unizg.hr (L.B.); mvucelja@sumfak.unizg.hr (M.V.); ktomljanovic@sumfak.unizg.hr (K.T.); jmargaletic@sumfak.unizg.hr (J.M.); 3 University Hospital for Infectious Diseases “Dr. Fran Mihaljević”, 10000 Zagreb, Croatia; ncetinic@bfm.hr (N.C.B.); szember@bfm.hr (S.Z.); 4 School of Dental Medicine, University of Zagreb, 10000 Zagreb, Croatia

**Keywords:** tick-borne encephalitis virus, Croatia, TBE incidence, TBE seroprevalence, biogeographic diversity, climate, precipitation, wildlife

## Abstract

Tick-borne encephalitis (TBE) is a common arbovirus infection in Croatia. The aim of the study was to analyse 17 years of data on TBE seroprevalence and acute TBE cases in correlation with winter temperature, precipitation and wildlife abundance to identify possible patterns that may be predictive indicators of TBE incidence. Clinical diagnosis of TBE was confirmed by determining IgM and IgG anti-TBE antibodies. Of the 19,094 analysed patients, 4.2% had acute TBE, significantly more often in older age (*p* < 0.001) and male gender (*p* < 0.001). Overall seroprevalence of TBE among the tested population was 5.8% and varied annually from 2.8% to 10.7%. The mean acute TBE incidence rate was 1.1/100,000 population with significant regional differences: 1.7/100,000 in the continental vs. 0.2/100,000 and 0.5/100,000 in the Mediterranean and Alpine regions, respectively. A particularly high incidence of 3.1/100,000 was recorded in northern Croatia. TBE displayed a seasonal pattern, peaking in June and July. Moderate negative correlations were observed between TBE acute cases and winter temperatures from December to February (r = −0.461; *p* = 0.062), relative rodent abundance (r = −0.414; *p* = 0.098) and yearly precipitation from one year before (r = −0.401; *p* = 0.123). The analysis showed that more acute TBE cases are recorded after a warmer winter and a negative correlation between the abundance of forest *Apodemus* sp. and the number of TBE cases in the same year.

## 1. Introduction

Tick-borne encephalitis (TBE), caused by the *Orthoflavivirus encephalitidis* virus, a neurotropic arbovirus from the genus *Orthoflavivirus*, family *Flaviviridae*, is a potentially life-threatening central nervous system infection prevalent in eastern, central and northern Europe, including Croatia [1]. The European subtype virus (TBEV-Eur) is the etiological agent of TBE in central Europe [1,2]. More than 10,000 human cases of TBE occur in Europe and Asia annually [3]. The yearly sum of reported TBE cases in 25 European Union (EU) and European Economic Area (EEA) countries in the period 2012–2016 ranged from 1950 to 3027. Lithuania and Czechia accounted for over a third (38.6%) of TBE cases during this period [4]. Reported cases in Croatia during the same period ranged from 6 to 45, and the average notification rate/100,000 inhabitants was 0.68, slightly higher than the EU/EEA average of 0.54 [4]. The majority of the reported TBE cases in Croatia occurred in a relatively small continental area between the Sava and Drava rivers, most notably in the Koprivnica-Križevci county, with an average annual incidence of 5.2/100,000 population [5]. More than 20 species of ticks belonging to several genera (*Ixodes*, *Dermacentor*, *Haemaphysalis*, etc.) have been found to carry TBEV, but two species are most important for virus circulation: *Ixodes ricinus* (dominant in Europe) and *Ixodes persulcatus* (dominant in Russia and Asia) [6]. Ticks occur in three post-embryonic life stages: larva, nymph and adult. All three stages feed on blood from vertebrate hosts. Non-feeding ticks of all stages tend to assume a static anticipatory position: questing. Larvae almost never climb more than 70 centimetres above ground to quest and tend to feed on smaller animals such as small mammals, birds and lizards. Adult ticks can climb up to 1.5 m and are therefore more likely to attach to larger animals like deer [6,7].

Small rodents are crucial for the maintenance of TBEV within the tick population. In Europe, mice and voles, especially the yellow-necked mouse (*Apodemus flavicollis*) and the bank vole (*Clethrionomys glareolus*), are considered the most important for TBEV transmission [6]. TBEV viremia in rodent hosts is relatively short-lived; therefore, non-viremic transmission is crucial for the maintenance of TBEV in ticks. Non-viremic TBEV transmission occurs during co-feeding: a naive larva can take up the virus by attaching and feeding near infected nymphs on the same host [6,8]. Large mammals, mainly the roe deer (*Capreolus capreolus*), act as mating sites and a food source for adult ticks, making them the main biotic factor driving tick abundance. The migration of deer is an important factor for the propagation of ticks to new areas [9].

As with other highly important zoonotic diseases, a multifaceted, integrated approach is required to formulate effective and economically viable measures against human infection and disease caused by TBEV. This is partly due to the fact that the dynamics of TBEV transmission are a result of interaction between several ecological and environmental factors: arthropod vector populations, zoonotic vertebrate hosts, geography, vegetation, climate and others [6]. Tick biology and TBEV transmission are clearly influenced by weather parameters such as temperature and humidity. Higher temperatures increase the rate of tick interstage development, potentially increasing tick density and extending the period in which ticks are actively searching for a host [10]. Mild winters and prolonged spring and autumn seasons positively affect tick population numbers [11]. Tick activity is also highly dependent on daylight duration and relative humidity (RH) levels. Ticks are sensitive to desiccation in prolonged dry conditions [12]. The seasonal synchronicity of larval and nymph life stages allows for the focal enzootic transmission of TBEV through co-feeding. Rapid autumn cooling causes larvae to enter diapause and continue questing in the spring along with nymphs [13]. In dry weather, quantified by air saturation deficit, nymphs tend to quest closer to the ground to avoid dehydration. This makes them more likely to co-feed with larvae on small mammals [9].

Human behaviour, including professional or recreational activities in nature, such as mushroom picking, plays a key role in the epidemiology of TBE by increasing exposure to infected ticks. However, the development of tick-borne disease is influenced by a complex interplay of various factors [14,15,16,17]. The influence of one parameter on the change in TBE incidence is not easy to define.

In order to establish the most effective programme for the prevention of TBE infection, it is necessary to identify risk areas, define them geographically and monitor them over time, ideally through standardised continuous surveillance [18]. This is especially important for countries with a high level of biogeographic diversity, which results in large differences in disease risk between close geographic areas, like in Croatia. The geographical distribution of TBE in Croatia varies significantly, and vaccination programmes should be more widely implemented when areas with higher TBE prevalence become better defined. Croatia currently has no formal vaccination programme for TBE, only vaccine recommendations for those at higher occupational risk for infection. EU-level standard case definitions and a reporting system have been established and updated, but they have yet to be fully and consistently adopted by individual member states [18]. Underreporting and underdiagnosis of TBE cases have been reported, which may negatively impact surveillance [18,19,20,21,22].

TBEV transmission to humans is known to occur within relatively small endemic foci, where the environmental requirements for the enzootic cycle of TBEV are met [6]. Endemic hotspots have been observed in the same locations for decades, but it has been shown that new ones appear over time in new locations, and some of the existing ones may disappear [23,24,25]. Climate change has been implicated as a driving factor in some of the observed shifts in the geographical distribution of tick activity and, potentially, TBEV endemicity [26,27]. A more precise analysis of local TBEV endemicity can be established directly through focused surveillance of TBE cases in humans and seroepidemiological studies or through surveillance of TBEV transmission via detection of TBEV in ticks or anti-TBE antibodies in small vertebrate hosts or domestic animals [28,29,30,31,32]. Monitoring vector density and abundance of specific vertebrate hosts may enable the development of risk prediction models for TBE while considering the deep complexity of all the factors influencing the TBEV enzootic cycle and the epidemiology of TBE [33,34,35].

The aim of the study was to present and analyse 17 years of data on TBE human cases in Croatia. In correlation with temperature, precipitation and wildlife abundance, we attempted to identify possible patterns in climate and environmental data that could be useful in predicting TBE risk. The aim was to analyse whether warmer winters and drier summers affect the survival and efficient reproduction of tick populations in the following season and whether precipitation, which can affect both tick biology and human behaviour—potentially leading to greater exposure to vectors—may be associated with the incidence of TBE cases.

## 2. Materials and Methods

### 2.1. Data Collection

#### 2.1.1. TBE Cases

The analysis included patients with suspected TBE with neurological symptoms or aseptic meningitis who were referred for anti-TBE antibody testing from 2002 to 2018 to the University Hospital for Infectious Diseases in Zagreb, Croatia, the leading national medical centre for infectious diseases. The diagnosis of TBE was based on clinical and epidemiological data and the determination of IgM and IgG anti-TBE antibodies. Commercially available IgM and IgG anti-TBE enzyme-linked immunosorbent assays (ELISA, FSME/TBE IgG/IgM ELISA, Virotech Diagnostics, Germany) were used according to the manufacturer’s instructions. Acute TBE cases were confirmed by the presence of TBE-specific IgM and IgG in the serum or seroconversion or a four-fold increase in TBE-specific antibodies in paired serum samples [36]. Individual clinical acute cases of TBE were used for the analysis. Patient age, sex, date of sampling and sample collection site, specified to the level of county, were included in the dataset. West Nile virus (WNV) infection was ruled out in all cases of TBE that occurred after the first reported human infection of WNV in Croatia in 2012 [37].

#### 2.1.2. Common Game and Rodent Abundance

Game data were obtained from a publicly available hunting record e-database of the Ministry of Agriculture [38]. Total numbers of shootings per year for roe deer (*Capreolus capreolus*), red deer (*Cervus elaphus*), wild boar (*Sus scrofa*) and common pheasant (*Phasianus colchicus*) were used for the analysis. The relative abundance of rodents was calculated as the number of trapped animals per number of trap nights. Rodents were trapped predominantly in lowland forests as part of routine rodent monitoring using snap traps, for which guidelines by Gannon et al. were followed [39]. Trapping was conducted in late spring (May or June) and/or in early autumn (September or October). As part of routine monitoring in forestry, only external characteristics (fur colouration and tail length) were used to identify rodents, and the following species or genus categories were used in the analysis: 1. *Clethrionomys glareolus* (bank vole), 2. *Microtus* voles, 3. *Apodemus agrarius* (striped field mouse), 4. *Apodemus* sp. (yellow-necked mouse (*A. flavicollis*) and wood mouse (*A. sylvaticus*)).

#### 2.1.3. Winter and Summer Temperatures and Precipitation

The temperature data for the City of Zagreb representing the continental regions with the largest number of reported acute TBE were analysed for the period 2002–2018. The analysis included the following temperature parameters: average monthly temperatures for December, January, February, June, July and August, as well as three-month average temperatures for the winter season (December, January, February) and the summer season (June, July, August). Monthly precipitation data (rainfall in mm) from 12 different meteorological stations scattered through continental Croatia were obtained. All weather data were acquired from the Croatian Meteorological and Hydrological Service (DHMZ).

### 2.2. Statistical Methodology and Analysis

Acute TBE case age groups were defined as follows: 0–4, 5–14, 15–24, 25–44, 45–64 and 65 years and older, and sexes were defined as male and female. Total population numbers for all six age groups and both sexes were obtained. Pearson’s chi-squared test for independence was used to determine whether the distribution of acute TBE cases was significantly different across the age groups and between sexes.

One-way ANOVA was conducted to assess the differences in the mean number of acute TBE cases across all 12 months for the period 2002–2018. A post-hoc analysis using the least significant difference (LSD) test was performed to identify specific months that differed significantly in their mean TBE case counts.

TBE incidence rates per 100,000 population were calculated for each year from 2002 to 2018 for Croatia as a whole and for each county individually. A map of Croatia was generated showing biogeographical regions: continental, Mediterranean and Alpine regions with ArcGIS^®^ 9.3. Incidences of TBE in individual counties are represented by the diameter of the circle positioned approximately at the administrative centre of each county.

The correlation between acute TBE cases and population numbers of rodents, common game species, winter temperature parameters and annual and mean monthly precipitation was tested with the Pearson’s correlation coefficient. Demographic data required for the calculations were obtained from the Croatian Bureau of Statistics [40,41]. All statistical analysis in this paper was conducted in Statistica Version 14.0.0.15 TIBCO Software Inc. (Palo Alto, CA, USA).

## 3. Results

**Figure 1:**
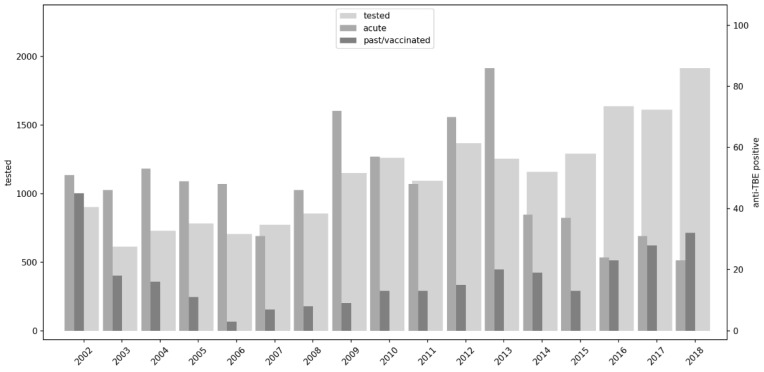
TBE cases from 2002 to 2018 in Croatia: total tested persons (*Y*-axis left), acute TBE and residual anti-TBE IgG antibodies (*Y*-axis right).

A total of 19,094 patients in the 17-year period from 2002 to 2018 were tested for IgM and IgG anti-TBE antibodies. Anti-TBE IgM and/or IgG antibodies were positive in 1103 (5.8%) subjects. Annual seroprevalence among the tested population ranged from 2.8% to 10.7%. A total of 810 patients had acute TBE, which accounted for 4.2% of tested and 73.4% of all positive patients. Residual anti-TBE IgG antibodies were found in 293 (1.5%) subjects. The mean number of acute TBE cases per year was 47.7 (SD ± 17.00). The annual number of acute TBE cases varied from 23 to 86 (range of 63) (Figure 1).

**Figure 2:**
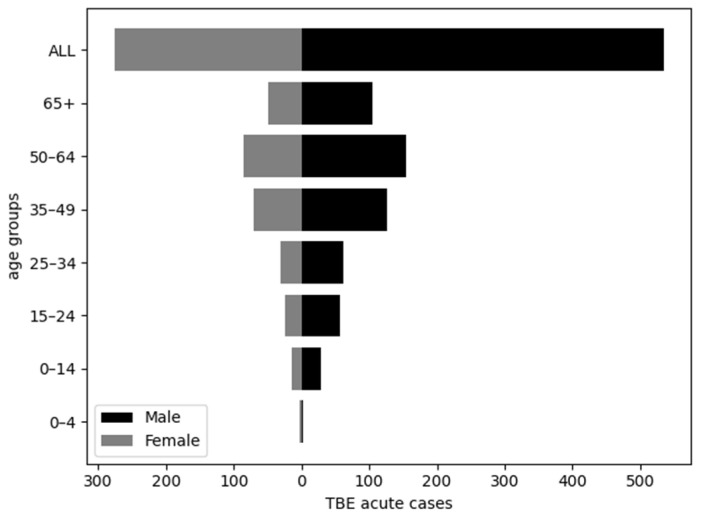
Age–sex pyramid chart for acute TBE cases, 2002–2018.

The number of acute TBE cases was statistically different between age groups (χ^2^ = 78.471, *p* < 0.001) and sex (χ^2^ = 64.479, *p* < 0.001). The highest proportion of acute cases was detected in the older age groups, especially in the age group 45–64 (37.8%). The male-to-female ratio of acute TBE cases was 1.9/1. Male patients accounted for 65.9% of acute cases (Figure 2).

**Figure 3:**
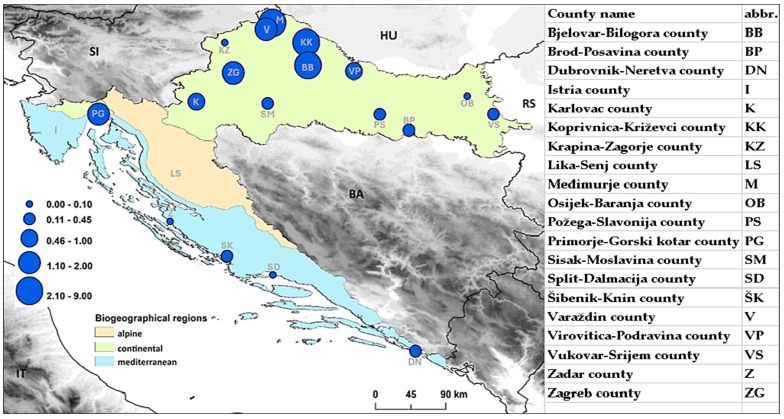
Biogeographical regions in Croatia with confirmed acute TBE cases per 100,000 population for each county for the 2002–2018 timeframe. Each circle and county abbreviation is placed approximately in the position of the county seats.

**Figure 4:**
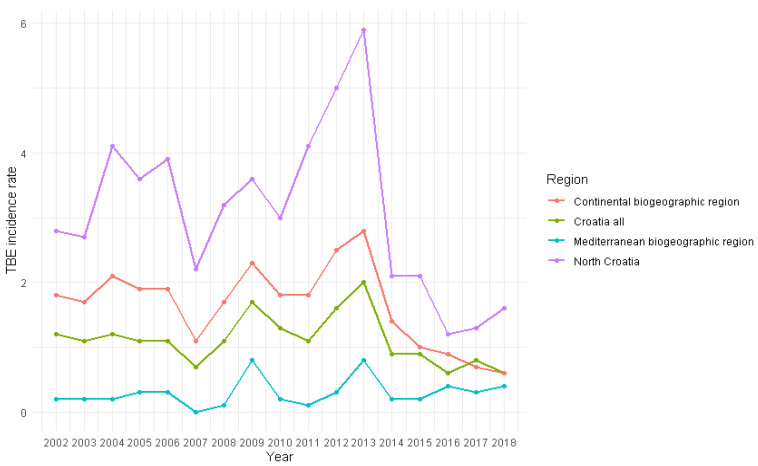
Acute TBE incidence rates per 100,000 population for each year, 2002–2018.

The overall incidence of TBE for the 2002–2018 timeframe was 1.12/100,000 population (SE 0.10). The highest incidence was recorded within the continental biogeographical region (1.65/100,000 population, SE 0.15) (Figure 3, green areas). Of the 810 acute TBE cases, 787 (97.16%) were in the continental biogeographical region, particularly in the North Croatia region (consisting of Zagreb, Koprivnica-Križevci, Varaždin, Međimurje and Krapina-Zagorje counties; 3.09/100,000 population, SE = 0.31) (Figure 3 and Figure 4).

**Figure 5:**
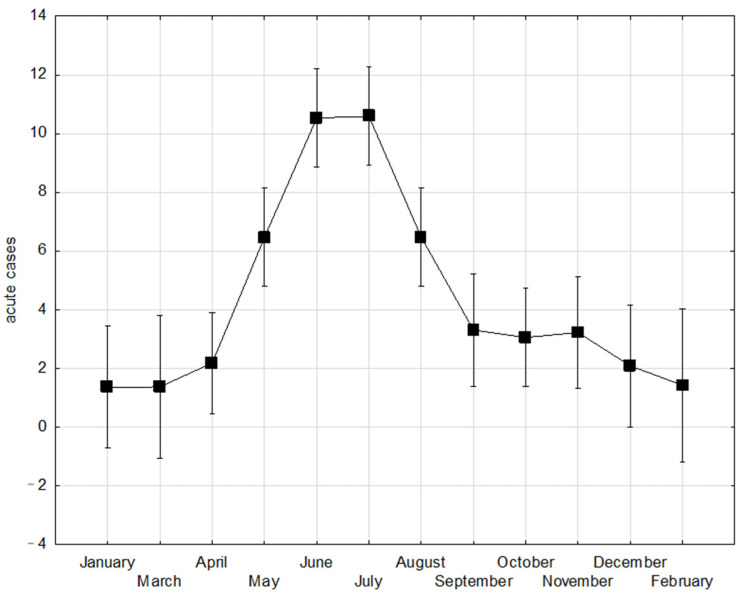
Distribution of acute TBE by month from 2002 to 2018 (vertical bars denote 0.95 CI).

Typical seasonality, with the highest numbers of acute TBE cases from May to August, was recorded. The ANOVA analysis of the variance of mean monthly TBE case numbers from 2002 to 2018 indicates a statistically significant difference in the means across the months (F (11, 152) = 13.674, *p* < 0.001). Post-hoc analysis reveals notable peaks in mean monthly TBE cases in July (10.59 ± SE 0.84) and June (10.53 ± SE 0.84). Conversely, the lowest number of acute TBE cases was observed during the winter months of December, January and February (Figure 5).

No significant correlation was found between all tested temperature parameters and the number of acute TBE cases both in Zagreb and throughout Croatia. Similar results were found when temperatures from the previous year were correlated with acute TBE cases (Table 1). However, a moderate negative correlation was observed between mean winter temperatures and acute cases of TBE for the whole of Croatia in the same year (December, January and February) (r = −0.495; *p* = 0.043).

**Figure 6:**
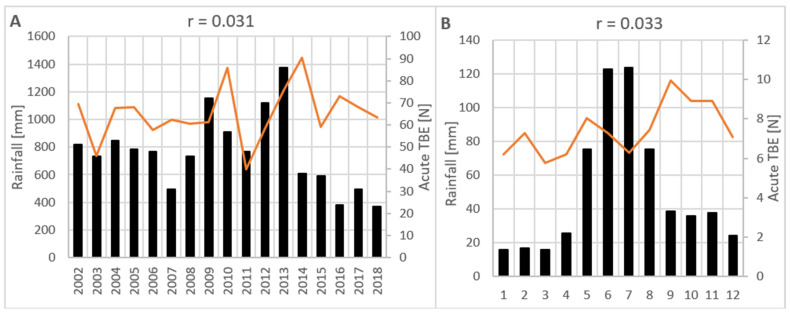
(**A**) Annual precipitation and number of TBE cases. (**B**) Mean monthly precipitation and number of acute TBE cases. r = Pearson’s correlation coefficient. Orange line = rainfall/precipitation; black bars = acute TBE.

For the entire period from 2002 to 2018, the total annual precipitation did not show any correlation with acute TBE cases (r = 0.031; *p* = 0.905) (Figure 6A). There was a moderate negative correlation found between annual precipitation from the previous year and the incidence of acute TBE cases (r = −0.401; *p* = 0.123). There was no significant correlation found between monthly precipitation and monthly acute TBE cases (r = 0.033; *p* = 0.637) for the entire period from 2002 to 2018 (Figure 6B). The same result was obtained in the correlation of monthly acute TBE cases and rainfall from the previous month (r = −0.025; *p* = 0.726).

**Figure 7:**
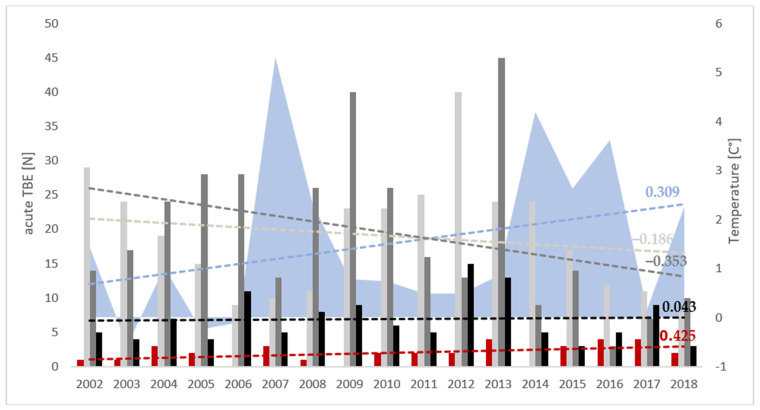
Display of mean winter temperatures (December–February; blue) and numbers of acute TBE cases for January–March (dark red), April–June (light grey), July–September (dark grey) and October–December (black) from 2002 to 2018. Each dashed line shows a correlation (r = Pearson’s correlation coefficient) of the same coloured data over time.

Over the period 2002–2018, there was a slight downward trend in TBE cases (r = −0.301; *p* = 0.240) (Figure 4). We recorded an increasing trend of acute TBE cases early in the year (January-March) (r = 0.425; *p* = 0.089). Spring and summer months showed a slight decrease in acute TBE cases (April-June: r = −0.186; *p* = 0.476; July-September: r = −0.353; *p* = 0.164) (Figure 7). During the analysed 17-year period, the mean winter temperature increased from 1.2 °C in the first nine years to 1.8 °C in the subsequent eight years (r = 0.309; *p* = 0.228) (Figure 7). Similarly, the mean summer temperature rose from 20.2 °C to 20.8 °C over the same timeframes (r = 0.279; *p* = 0.278).

The correlation results between yearly game abundance for each of the four game species (roe deer, red deer, wild boar and common pheasant) and acute TBE cases showed no statistical significance (Table 2). Similar results were obtained when game abundance data were correlated with acute TBE cases from one year after (Table 2).

**Figure 8:**
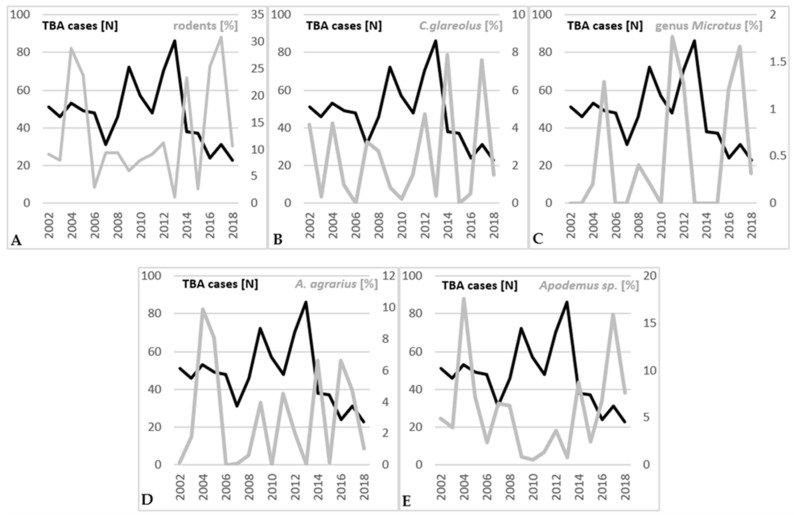
Relative rodent abundance (grey line) and yearly TBE cases (black line) for 2002–2018. (**A**) all captured rodents; (**B**) *Clethrionomys glareolus* (bank vole); (**C**) genus *Microtus*; (**D**) *Apodemus agrarius* (striped field mouse); (**E**) *Apodemus* sp. (yellow-necked mouse (*A. flavicollis*) and wood mouse (*A. sylvaticus*)).

The majority of the rodents captured belonged to the genus *Apodemus* (74.6%), followed by the bank vole (*Clethrionomys glareolus*), which comprised 19.9% of the total, and species from the genus *Microtus*, which were the least common at 5.6% (Figure 8). Within the genus *Apodemus*, 64.1% of the captures were classified as *Apodemus* sp., of which 36.2% were identified as *A. flavicollis*. A total of 27.9% could not be identified and may represent either *A. flavicollis* or *A. sylvaticus*. The other 35.9% of the captures from the genus *Apodemus* were identified as the striped field mouse (*A. agrarius*). Rodent abundance varied by species and genus, as shown in Figure 8, but no significant correlation was observed between rodent abundance and the incidence of acute TBE cases in the same year, one year later, or two years later (Table 3). Nevertheless, a moderate negative correlation was found between relative rodent abundance and TBE cases in the same year (r = −0.414, *p* = 0.098), as well as with the *Apodemus* species (*A. flavicollis* and *A. sylvaticus*) in the same year (r = −0.442, *p* = 0.075) (Table 3).

## 4. Discussion

Despite its small area, Croatia is extremely biogeographically diverse, which is reflected in the heterogeneous endemicity of TBE. The largest known TBE hotspots are located between the Sava and Drava rivers and in Gorski Kotar (Primorje-Gorski Kotar county) [42,43]. Our data are consistent with previous analyses, with an incidence of 1.64/100,000 population in the period from 2002 to 2018 for the continental part of Croatia. Most notably, within the continental biogeographic region, the highest numbers of patients were recorded in the counties of northern Croatia: Koprivnica-Križevci, Bjelovar-Bilogora and Međimurje (8.2, 4.1 and 4.0/100,000 inhabitants, respectively). These areas represent a southeastern extension of the Slovenian TBE endemic areas, with the southernmost border approximately corresponding to the 45th parallel [42,43]. The overall incidence of TBE in Croatia in the period from 2002 to 2018 was 1.12 per 100,000 population. In historical context, Croatian yearly TBE incidence has regularly exceeded 2.0/100,000 population during the 1970s, peaking at 3.4/100,000 population in 1972, which is significantly more than the highest incidence years from our analysed period [44]. Although Slovenia always had a markedly higher TBE incidence than Croatia, the variations in incidence throughout the 1970s and 1980s largely coincided [43]. In comparison with the EU/EEA data (reporting at the EU/EEA level started in 2012), the incidence in Croatia, according to our recalculated data for the period 2012–2018, was 1.05/100,000 population and is slightly higher than the average EU/EEA TBE incidence (0.73/100,000 population), but it is significantly lower than that in neighbouring Slovenia (6.73/100,000 population) [45].

TBE was first reported in Croatia in 1953 and has been a notifiable disease since 1987 [43,46]. TBE reporting is mandatory; the European Centre for Disease Prevention and Control (ECDC) case definitions are used, and it is usually tasked to the physician treating the patient [47]. There is heterogeneity in the surveillance and reporting systems between EU countries, which somewhat hinders the inter-comparability of national TBE data [18,48]. TBE is assumed to be an underreported disease, and the true burden of TBE in the EU is underestimated [18]. This study is based on the results of diagnosis and treatment from a tertiary medical centre for infectious diseases where the largest number of patients with central nervous system infections, including TBE, are diagnosed and treated. In seventeen years of follow-up, acute TBE was diagnosed in 810 patients, while only 466 of them were officially reported, which further confirms the problem of insufficient reporting. All presented results were interpreted individually for each subject in accordance with appropriate epidemiological and clinical data to confirm acute TBE diagnosis.

The TBE distribution over age groups was similar to the EU/EEA-wide data reported by ECDC and was the highest in the 45–64 age group (37.78% and 37.98%, respectively). A slightly higher proportion of acute TBE cases was reported in the 15–24 age group compared to ECDC data (9.9% vs. 7.9%), as well as a higher male-to-female ratio (1.9/1 vs. 1.5/1, respectively) [48]. This could indicate a more pronounced difference in the infection risk between sexes in Croatia, possibly due to occupational exposure.

The incidence of TBE has a regular seasonal pattern that was confirmed in our analysis. Most cases were detected in the period from April to September, with the most prominent peaks in June and July. The annual TBE upsurge throughout the observed period was statistically significant for the months of May, June, July and August. A small TBE increase was observed during autumn (October and November), creating a discrete bimodal pattern. In the EU/EEA-wide analysis, TBE cases usually peaked in July and August, and a bimodal pattern also occurs [48,49]. Tick activity is typically highest in late spring and sometimes can have bimodal dynamics, with a minor second peak in autumn [50,51]. This smaller autumn increase could be due to an increase in precipitation, which provides the moisture to support tick host-seeking behaviour [16]. During the warmer months, human activity also increases and leads to an increased risk of tick bites further fuelling the annual increase in TBE incidence [17]. The characteristic biphasic clinical course of TBE should be considered: after the tick bite and incubation phase (median of 8 days), a non-specific prodromal phase occurs, lasting approximately 5 days, which, after a variable asymptomatic period (1–21 days), is followed by the second disease stage with CNS involvement, which is when the diagnosis is usually made [5]. The increase in TBE cases is accompanied by a seasonal increase in tick activity, but with a lag of 2–4 weeks, approximately accounting for the time between infection and diagnosis [5].

The effect of climate change on the distribution of ixodid tick activity and the extent of transmission of TBEV and other vector-borne agents has attracted much attention during recent decades. A warmer climate potentially means an earlier onset and longer duration of tick activity, and milder winters allow the propagation of tick populations into the next season [52]. The progressive increase in temperatures has resulted in the expansion of tick habitats into areas previously deemed tick-free, e.g., higher altitudes in mountainous regions and the subarctic areas of northern Europe. In a 1981–1983 field study in the Czech Republic, *Ixodes ricinus* populations were stable up to 700 m above sea level. In a later study from 2002, tick activity up to 1100 m was confirmed. In Sweden, a trend of tick habitat expansion towards the north was observed [26,27,28]. Milder winters, which are expected to occur more often due to climate warming, allow tick propagation to be maintained for a longer part of the year, which can lead to an increase in their numbers throughout the year [53,54]. Analysing data over a 38-year period, a Swedish study showed a correlation between a combination of temperature variables, including two consecutive milder winter seasons, and an increased incidence of TBE [54]. Similarly, our analysis also showed the influence of warmer winters on the numbers of TBE cases as well as an increase in cases early in the year (January–March). A milder winter increases the chance of survival of cold-sensitive hibernating larvae and their melting into nymphs during the warmer season in the same year. Those nymphs can then hibernate during the coming winter and become active the following spring [54]. The winter of 2006/2007 was one of the warmest ever recorded in Zagreb, followed by another warm winter, which preceded the increase in TBE incidence in 2009.

Observing the temporal dynamics of TBE incidence during the analysed period, two peaks were recorded: in 2009 and 2013, and a decline in incidence in the period 2015–2018 [47]. Neighbouring Slovenia also reported increased incidence in 2009 and 2013 [55]. A large increase in the number of cases in Slovenia and several other European countries was observed in 2006 but was less pronounced or absent in other countries, including Croatia [16,55,56]. According to our data, the state-level TBE incidence in 2006 was the same as in 2005 (1.1/100,000 population), and slightly lower than in 2004 (1.2/100,000 population). At a local level, in northern Croatia, it did rise in Koprivnica-Križevci county to 11.8/100,000 population, the second highest incidence there in our tested period, and in Varaždin county, while it simultaneously dropped in Međimurje and Bjelovar-Bilogora counties to values lower than in 2004 and 2005. Northern Croatia did not have a sharp peak in incidence in 2006 or the previous four years, but rather a plateau of relatively high incidence that was followed by a drop in 2007. Interestingly, Primorje-Gorski Kotar county had relatively low TBE incidence during 2002–2006 but peaked sharply in 2009 to 5.4/100,000 population, strongly contributing to the 2009 incidence peak in Croatia. The 2013 peak was again largely driven by the major increase in TBE cases in northern Croatia. The incidence in Primorje-Gorski Kotar also increased, but not as strongly as in 2009. Looking at this, it does seem that the dynamics of TBE hotspots of northern and Pannonian Croatia when compared to Primorje-Gorski Kotar have a certain level of eco-epidemiological independence. Virovitica-Podravina county, located in Pannonian Croatia on the southern bank of the Drava River, yielded no acute TBE cases in our analysis from 2002 to 2011, and then 9 cases occurred in the period from 2012 to 2018. We are not sure whether this is the result of the activation of a TBE focus within this county or due to reporting issues. The incidence dynamics of TBE in Croatia roughly align with those of neighbouring countries Slovenia and Hungary after 2007, including a notable decrease in TBE incidence starting in 2014 and extending into the COVID-19 pandemic years. In central Europe and the Baltics, the incidence has mostly stagnated or increased during this period, while in Scandinavia, most notably Norway and Finland, it has increased sharply [55]. Randolph et al. found that the weather-induced changes in tick abundance alone did not account for the aforementioned increase in TBE cases in 2006. The variance in incidence in 2006 between countries, despite similar weather patterns, was probably mostly due to factors associated with human behaviour, like outdoor recreation and activities like mushroom picking and the harvest of wild berries [56]. Daniel et al. concluded that in the Czech Republic, the peak of TBE incidence in 2006 was partly related to an unusually rainy August, which stimulated tick host-seeking activity in late summer/early autumn [16].

Precipitation is possibly related to the occurrence of TBE in humans, as it affects the biology of components of the TBEV enzootic cycle as well as human behaviour that leads to greater exposure to ticks [56,57]. Several authors have presumed the influence of rainfall on TBE incidence and have included precipitation data in prediction models [16,56,57,58]. Danielova et al. found that during the TBE season, a period of increased rainfall is often followed by a period of increased TBE incidence with a latency corresponding to the incubation period of TBE. It is argued whether the influence of increased relative humidity on the activity of ixodid ticks combined with increased human activity after the end of the rainy season can result in a short-term increase in TBE cases [57]. Haemig et al. found a correlation between TBE incidence and precipitation in December of the previous year, but not in other months. A tentative theory for this correlation could be the protective effect of snowfall on the survival of hibernating ticks [58]. The protective effect of snow cover on the winter survival of ticks has been previously hypothesised, but the data are limited [59]. If we expand on this, in Croatia, the winter of 2012/2013, marked by heavy snowfall, was followed by an exceptionally warm period in late April and early May, which coincided with the highest recorded annual incidence of TBE in this study. Our data did not prove a relationship between rainfall and TBE incidence, but this does not mean that it does not exist and should not be further studied.

A network of ecological relationships is fundamental for effective local TBEV transmission, and it involves many animal species. Large vertebrate hosts, especially roe deer, are a major source of blood meal for ticks and have a positive effect on tick numbers; but, being dead-end hosts, they are not directly involved in TBEV transmission. In extremely large numbers, deer may divert ticks away from TBE transmission-capable hosts such as mice and voles, potentially reducing the TBEV burden rather than increasing it [60,61,62]. We found no correlation between deer numbers and acute TBE cases. However, we found a moderate negative correlation between rodent abundance, as well as the abundance of the *Apodemus* species, and TBE incidence. This is somewhat confusing, as *Apodemus* mice play a large role in TBEV transmission. A potential explanation for this could be that when *Apodemus* mice are highly abundant, ticks prefer to feed on them rather than seek alternative hosts, like humans. The possible dilutive effect of biodiversity, including large abundances of various rodent species, many of which are less competent TBEV reservoirs, similar to what has been observed in Lyme borreliosis, should also be taken into account [63,64,65]. The negative correlation we found between forest rodents and TBE acute cases in the same year should be subjected to a more detailed analysis because the number of forest rodents tends to decline in years after the population outbreaks associated with acorn and beech mast. Forest rodent monitoring could be a potential tool for predicting possible TBE outbreaks [66,67].

We are aware of the limitations of this study and that the analysed period of seventeen years is still not enough to draw major conclusions, but we believe that it contributes to the improvement of knowledge about TBE, especially since there is a lack of similar research in Croatia. There is a notable discrepancy between the number of acute TBE cases in our dataset and the number of cases reported to ECDC from 2012 to 2018 (309 and 178, respectively). The greatest issues in case reporting in individual counties are short-term migrations: a person diagnosed with TBE at one site could have been infected within a different geographical region. Patients from counties neighbouring a larger medical centre, e.g., Zagreb, often gravitate toward that centre and register there, which results in an overflow of the case numbers. Also, individual patients may have been tested in other laboratories and, therefore, are not included in our dataset, but we nevertheless recorded a higher number of acute TBE cases than officially reported, and despite this, the regional distribution remains. It is known that the complex multifactorial interaction between the tick and the reservoir, with the influence of various environmental factors, makes the assessment of the possible effect of a single variable on the incidence of TBE difficult. Although we used temperature data from a single location (Zagreb), we believe that due to its central position, it is representative of the continental region and sufficient for a preliminary analysis. Further research should certainly include more detailed local data analysis. In Croatia, there are well-known endemic areas between the Sava and Drava rivers and in the Gorski Kotar northeast of the Istrian peninsula, areas which differ in terms of fauna. Therefore, the role of the number of animals, which we analysed for the entire country, even though most hunting grounds were related to continental areas with the highest number of TBE, would be worth investigating in narrower local areas.

## 5. Conclusions

The geographical distribution of TBE in Croatia is highly variable within the continental region itself and even more so when the Mediterranean and Alpine biogeographic areas are included in the comparison. Consequently, the generalised data in ECDC reports may deviate significantly from reality for certain regions. Regional data should be as accurate as possible because they have an impact on public health and national and international efforts to develop strategies to assess, manage and predict the TBE risk at the local level based on incidence reports. The analysis over a 17-year period showed the influence of winter temperatures on human TBE cases in Croatia, linking warmer winters with an increase in TBE incidence. There was also a negative correlation between the abundance of forest *Apodemus* sp. and TBE cases in the same year. In order to enable modelling and support a robust One Health approach, continuous long-term monitoring of epidemiological, environmental and meteorological data is needed to define local high-risk areas in which targeted preventive measures can be implemented.

## Figures and Tables

**Table 1:** Correlation between acute TBE cases (Zagreb County and the whole of Croatia) and the mean winter and summer temperatures of Zagreb County for 2002–2018.

Period Temperature	December	January	February	Winter (December–February)	June	July	August	Summer (June–August)
ZAGREB COUNTY acute TBE cases in the same year	r * = −0.085	r = −0.321	r = −0.307	r = −0.391	r = −0.086	r = 0.290	r = 0.304	r = 0.219
	*p* = 0.744	*p* = 0.210	*p* = 0.231	*p* = 0.121	*p* = 0.743	*p* = 0.258	*p* = 0.236	*p* = 0.397
ZAGREB COUNTY acute TBE one year after	r = 0.009	r = 0.181	r = −0.202	r = −0.021	r = 0.061	r = −0.181	r = 0.270	r = 0.118
	*p* = 0.973	*p* = 0.487	*p* = 0.437	*p* = 0.936	*p* = 0.817	*p* = 0.485	*p* = 0.294	*p* = 0.653
CROATIA acute TBE cases in the same year	r = −0.215	r = −0.352	r = −0.374	r = −0.495	r = −0.254	r = −0.012	r = 0.105	r = −0.041
	*p* = 0.406	*p* = 0.166	*p* = 0.139	*p* = 0.043 *	*p* = 0.326	*p* = 0.963	*p* = 0.690	*p* = 0.875
CROATIA acute TBE one year after	r = −0.094	r = 0.140	r = −0.369	r = −0.172	r = 0.105	r = −0.328	r = 0.275	r = 0.095
	*p* = 0.720	*p* = 0.591	*p* = 0.144	*p* = 0.510	*p* = 0.688	*p* = 0.198	*p* = 0.284	*p* = 0.718

* r = Pearson’s correlation coefficient.

**Table 2:** Correlation of annual acute TBE cases and most common game species population numbers for 2002–2018.

Game Species	Roe Deer		Red Deer		Wild Boar		Common Pheasant	
	r *	*p*-Value	r	*p*-Value	r	*p*-Value	r	*p*-Value
Acute TBE	−0.208	0.422	−0.334	0.189	−0.248	0.337	−0.354	0.163
Acute TBE one year after	−0.206	0.443	−0.186	0.488	−0.280	0.293	−0.251	0.347

* r = Pearson’s correlation coefficient.

**Table 3:** Correlation (r = Pearson’s correlation coefficient) of annual acute TBE cases (same year, one year after and two years after) and relative rodent abundance (%) for 2002–2018.

	Rodents (All)	*Clethrionomys glareolus*	Genus *Microtus*	*Apodemus agrarius*	*Apodemus* sp. (*A. flavicollis* & *A. sylvaticus*)
Acute TBE	r * = −0.414	r = −0.188	r = −0.157	r = −0.140	r = −0.442
	*p* = 0.098	*p* = 0.471	*p* = 0.547	*p* = 0.592	*p* = 0.075
Acute TBE one year after	r = −0.207	r = 0.014	r = 0.231	r = −0.042	r = −0.244
	*p* = 0.443	*p* = 0.958	*p* = 0.389	*p* = 0.878	*p* = 0.363
Acute TBE two years after	r = −0.356	r = −0.117	r = 0.055	r = −0.325	r = −0.228
	*p* = 0.193	*p* = 0.677	*p* = 0.845	*p* = 0.237	*p* = 0.413

* r = Pearson’s correlation coefficient.

## Data Availability

The raw data supporting the conclusions of this article will be made available by the authors on request.
